# The Role of Radiation Induced Injury on Lung Cancer

**DOI:** 10.3390/cancers9070089

**Published:** 2017-07-12

**Authors:** Stephanie Puukila, Christopher Thome, Antone L. Brooks, Gayle Woloschak, Douglas R. Boreham

**Affiliations:** 1 Department of Biology, Laurentian University 935 Ramsey Lake Road, Sudbury, ON P3E 2C6, Canada; cthome@nosm.ca (C.T.); dboreham@nosm.ca (D.R.B.); 2 Department of Medical Sciences, Northern Ontario School of Medicine, 935 Ramsey Lake Road, Sudbury, ON P3E 2C6, Canada; 3 Department of Environmental Science, Washington State University, 2710 Crimson Way, Richland, WA 99354, USA; tbrooks@tricity.wsu.edu; 4 Department of Radiology, Northwestern University, 300 E. Superior Street, Chicago, IL 60611, USA; gayle.woloschak@gmail.com; 5 Bruce Power, 177 Tie Road, Tiverton, ON N0G 2T0, Canada

**Keywords:** inhaled radionuclides, radiation-induced lung cancer, dose rate, cell killing

## Abstract

This manuscript evaluates the role of cell killing, tissue disorganization, and tissue damage on the induction of lung cancer following low dose rate radiation exposures from internally deposited radioactive materials. Beagle dogs were exposed by inhalation to ^90^Y, ^91^Y, ^144^Ce, or ^90^Sr in fused clay particles. Dogs lived out their life span with complete pathology conducted at the time of death. The radiation dose per cell turnover was characterized and related to the cause of death for each animal. Large doses per cell turnover resulted in acute death from lung damage with extensive cell killing, tissue disorganization, chronic inflammatory disease, fibrosis, and pneumonitis. Dogs with lower doses per cell turnover developed a very high frequency of lung cancer. As the dose per cell turnover was further decreased, no marked tissue damage and no significant change in either life span or lung cancer frequency was observed. Radiation induced tissue damage and chronic inflammatory disease results in high cancer frequencies in the lung. At doses where a high frequency of chromosome damage and mutations would be predicted to occur there was no decrease in life span or increase in lung cancer. Such research suggests that cell killing and tissue damage and the physiological responses to that damage are important mechanisms in radiation induced lung cancer.

## 1. Introduction

Radiation induced cancer has been related to radiation dose following single acute radiation exposures [1,2]. The dose–response relationship used by these groups suggests that each ionization increases the cancer risk in a linear fashion (Linear-No-Threshold Hypothesis (LNT)). According to the LNT Hypothesis, all levels of radiation should be considered as harmful. Decreasing the total dose, dose rate, or using dose fractionation however has a marked effect on the frequency of radiation induced cancer and a Dose, Dose-rate Effectiveness Factor (DDREF) has been established by national and international groups (1.5 NRC and 2.0 ICRP) to correct for this observation. These values have been questioned and postulated to be much higher [3,4,5].

The role of cell and molecular changes in the development of cancer such as mutations and chromosome aberrations have been viewed as a critical step in the pathway from normal cells to cancer. High dose rate exposure has been shown to lead to chromosomal aberrations and increased cell killing via apoptosis that leads to increased cell turnover [6]. Therefore, when determining radiation induced risk, cellular repair and turnover time must be considered. Cells that take longer to divide will be impacted differently than rapidly dividing cells exposed to the same dose rate. For example, bone marrow cells, with a turnover time of 3.6 days, would receive 1.0 hit/cell turnover when exposed to 0.1 Gy/year. Chromosome damage increases as a function of dose rate in this tissue [7]. Conversely, hepatocytes or blood lymphocytes, with a turnover time of years, could receive 100 hits/cell turnover when exposed to the same dose rate [6] and damage increases as a function of total dose in these cell types [8]. For internally deposited radionuclides we believe dose rate it is not appropriate to calculate average dose rate by dividing the total dose by time of appearance of the cancer [9,10]. This metric does not reflect energy deposition as a function of cell turnover, tissue damage, inflammatory disease, or cell and molecular damage induced per unit of time. Thus, an accurate prediction of risk must use a metric associated with the biology of the lung. The metric selected in this manuscript is the radiation dose per cell turnover which is a more accurate reflection of lung damage and lung cancer risk.

Review of the influence of dose rate on the key events in the critical pathways for the changes needed to take a normal cell to a cancerous cell have been evaluated and marked dose-rate effects found for most of these key events [6,11]. This manuscript is designed to evaluate the role of dose per cell turnover, tissue disorganization, and the breakdown of cell/cell and cell/tissue communication [12,13] increased cell turnover [14], cell killing [15,16], and chronic inflammatory disease [17,18] on lung cancer induction. These effects show a threshold for dose-rate below which dose or dose-rate have minimal impact on cancer incidence in this dog model.

## 2. Results

### 2.1. Half-Life and Dose per Cell Turnover

The time to deliver 95% of the total dose and the dose per cell turnover was calculated for each individual dog and radionuclide and the average of each is shown in Table 1. The biological half-life for each radionuclide is about 600 days since the aerosols of all radionuclide were delivered in fused clay. The effective half-life of ^90^Sr with the longest physical half-life would be similar to the biological half-life. The effective half-life and dose rate from ^144^Ce and ^91^Y would be influenced by both the physical and biological half-life. The short physical half-life of ^90^Y dominated the time when 95% of the dose was delivered. This results in the total dose of ^90^Y being delivered in a single cell turnover and thus a very high dose per turnover.

### 2.2. Dose Rate and Time to Death

**Figure 1:**
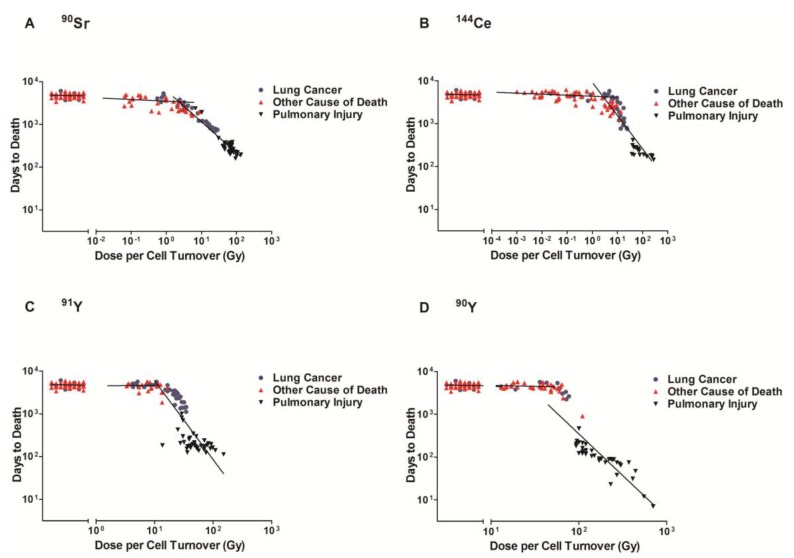
Cause of death of Beagle dogs exposed to radionuclides. The cause of death of dogs exposed to ^90^Sr (**A**), ^144^Ce (**B**), ^91^Y (**C**), and ^90^Y (**D**) in fused aluminosilicate particles was measured as a function of average dose per cell turnover. Transformed dose/cell turnover were plotted against days to death following inhalation. The cause of death is represented by a data point for each individual dog. Lung Cancer (●) Other Causes of Death (▲) and Pulmonary Injury (▼), Lines represent three linear regression analysis: control, area with limited life shortening (not significantly different than control) and significant life shortening (*p* < 0.05). At high doses early deaths were all related to pulmonary injury, lower doses resulted in longer survival and a high frequency of lung cancer, further lowering of the dose per cell turnover did not produce a significant decrease in life span or an increase in lung cancer.

The time after exposure to death and the cause of death were plotted against dose per cell turnover on a log-log graph. The dose/cell turnover was explained in the methods section. Figure 1 illustrates the cause of death related to the dose per cell turnover for each of the radionuclides (A ^90^Sr, B ^144^Ce, C ^91^Y, D ^90^Y). The order of appearance is related to the half-life of the radionuclide with the radionuclide with the longest half-life (^90^Sr) first and the shortest half-life (^90^Y) shown last. This figure illustrates that for all the radionuclides there was the same pattern. At very high dose rates per cell turnover most of the animals died from pulmonary injury within a year after inhalation. As the activity and dose per cell turnover decreased the life span increased to between five and nine years and the lung cancer incidence increased. With further decrease in dose per cell turnover below the level to cause life shortening no significant increase in lung cancer could be detected.

### 2.3. Dose Rate and Dose Response Functions

**Figure 2:**
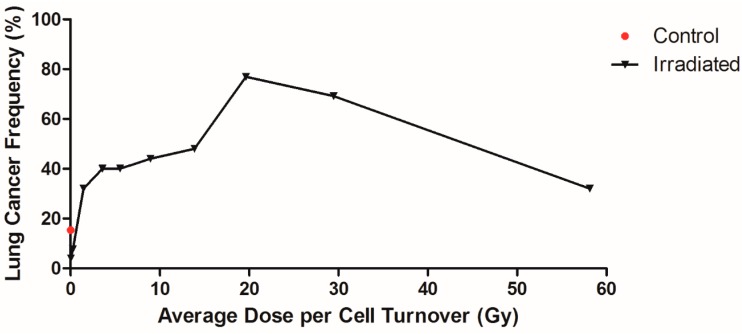
Lung cancer frequency of Beagle dogs exposed to radionuclides. Lung cancer frequency of dogs exposed to of ^90^Sr, ^144^Ce, ^91^Y, and ^90^Y in fused aluminosilicate particles was plotted against dose per cell turnover. The control dogs were plotted at the zero point with a lung cancer frequency of 15% and exposed dogs were split into 10 groups, 25–26 dogs per group, regardless of radionuclide. Each data point reflects the mean dose/cell turnover and cancer frequency for each group. The highest and lowest dose/cell turnover were not significantly different from control, while all other dose rates had significantly higher cancer frequencies (*p* < 0.05).

It is important to determine the dose/cell turnover vs. cancer response function. To do this, the animals were divided into groups as a function of dose per cell turnover and the frequency of lung cancer plotted. There were 52 control animals and 382 animals that inhaled the radioactive materials. The 129 animals that died of pulmonary injury were removed from the analysis since the survival time in this group was not long enough to induce lung cancer. The remaining 253 animals were divided into 10 groups with 25–26 animals per group and the frequency of cancers per group plotted as a function of dose per cell turnover. In this grouping, the radionuclides were not separated, since they are all low LET beta-gamma emitters, but all the animals exposed by inhalation were grouped. The results of this analysis are shown plotted in Figure 2. The control dogs group had a cancer frequency of 15%. Cancer frequency in the lowest and second lowest dose/cell turnover group was significantly less than control (*p* < 0.05). In the higher dose rate groups, cancer frequency reached 77% at an average dose rate of 19.65 Gy/cell turnover. The highest group had a cancer frequency of 32%. The majority of dogs in this group were exposed to ^90^Y, where most dogs died due to pulmonary injury and did not live long enough to develop lung cancer.

### 2.4. Influence of Dose per Cell Turnover

**Figure 3:**
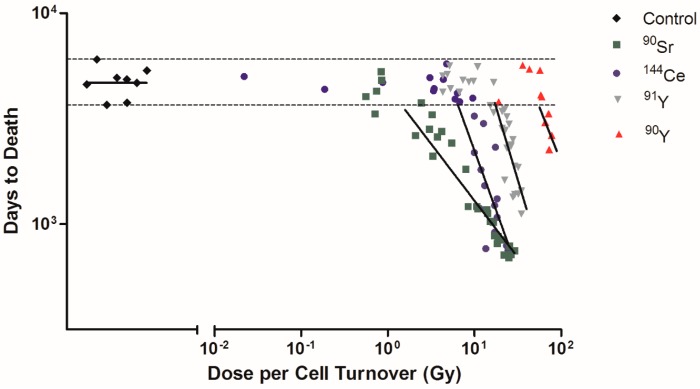
Dose per cell turnover was related to only the Beagle dogs that died due to lung cancer. Dose per cell turnover for ^90^Sr, ^144^Ce, ^91^Y, and ^90^Y in fused aluminosilicate particles was calculated as dose per cell turnover. Transformed dose per cell turnover were plotted against days to death following inhalation of particles for only the dogs that died due to lung cancer. Horizontal dotted lines indicate dogs that died due to spontaneous lung cancer that was not due to radiation exposure and were not included in linear regression analysis. Lines represent linear regression analysis of each radionuclide, where fit difference ranged from −0.54 to −1.38 (control was not significantly different from 0).

Among Beagle dogs that died of lung cancer, the dose rates of ^90^Sr, ^144^Ce, ^91^Y, and ^90^Y were calculated as dose per cell turnover (Figure 3). Exposed dogs that died of lung cancer after living a normal life span are shown on the figure as horizontal dotted lines. These were not included in the linear regression analysis as these dogs lived a normal life span and it is not possible to determine if the lung cancers were radiation induced or spontaneous. ^90^Sr has the lower dose per cell turnover required to produce cancer (less than 1 Gy) while ^90^Y has a higher dose (>20 Gy). The order of effectiveness for dose per cell turnover in producing cancer dependent on the effective half-life is ^90^Sr > ^144^Ce > ^91^Y > ^90^Y (Table 1). The lower dose per cell turnover makes it possible to not have fewer cells killed per cell turnover and to have a much larger total population of cells exposed. Only cells present at the time of the inhalation of the ^90^Y are exposed to very high dose rates while many generations of lung cells are exposed following ^90^Sr exposure. As this figure shows exposure to ^90^Y is much like a single acute exposure and all the dose is delivered in a single cell turnover. Killing of transformed cells by this exposure pattern is much higher and the induction of tissue changes needed to produce cancer are not as effective as exposure over longer time periods. The longer lived radionuclides exposed the cells over many cell divisions, the dose per cell turnover was lower, the repair of lethal damage greater, and more ‘transformed’ cells would survive and a much larger cell population was put at risk for cell transformation. Following these more protracted high dose exposures the tissue disorganization, breakdown of cell/cell and cell/tissue communication, and the production of a tissue environment which favors cancer development was present. Cancer induction was increased in this grossly modified tissue environment. The other interesting observation is that the time of onset of the cancers is earlier for the long lived radionuclides again suggesting that the tissue conditions needed to promote cancer are produced by lower dose rates delivered over a longer period of time. This suggests that the cancer induction is more a function of the tissue response and not a single cell response.

**Figure 4:**
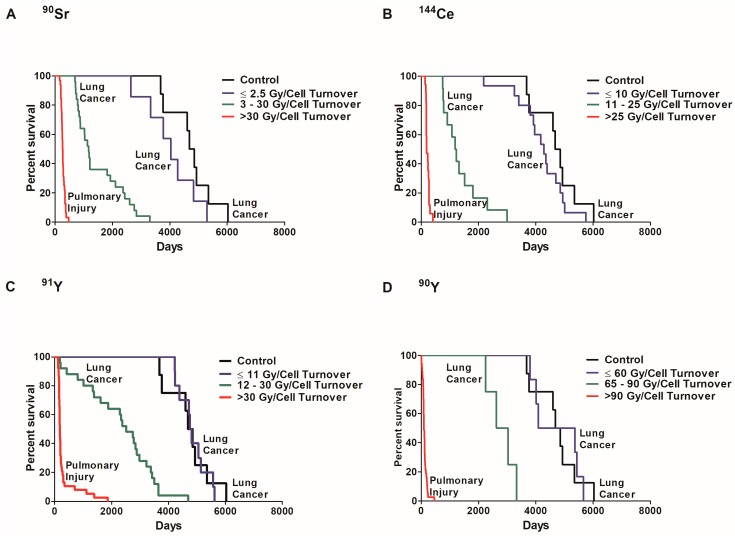
A Kaplan–Meier plot of dose per cell turnover on survival is shown along with the cause of death for each group. The percent survival of exposed and control dogs was plotted against days to death following inhalation of ^90^Sr (**A**), ^144^Ce (**B**), ^91^Y (**C**), and ^90^Y (**D**) in fused aluminosilicate particles of dogs that had lung cancer or pulmonary injury. Three distinct dose groups represent Gy/cell turnover: no significant life shortening or increased cancer frequency compared to control, significant life shortening with a very high lung cancer frequency and acute deaths (*p* < 0.05). Such data support the need for non-linear dose response functions and a change of paradigms toward total tissue responses and away from a single cell response.

In order to examine the role of dose per cell turnover on lung cancer risk, the percent survival of dogs that died of lung cancer or injury was investigated in exposed and control dogs (Figure 4). Dose response can be separated in three significantly different groups (*p* < 0.05) based on survival. The first group is exposed dogs that died of pulmonary injury where survival was significantly less than all other groups (*p* < 0.05). These dogs received a high initial lung burden due to high dose rates. The second group is exposed dogs that died of lung cancer. These dogs received a lower initial lung burden and survived longer than the first group but survival was significantly less than the controls (*p* < 0.05). The third group received radiation at a low dose rate, died of lung cancer, but lifespan was not significantly different from control dogs (*p* < 0.05). The dose per cell turnover required for the different group classifications were different for each radionuclide. A dose rate of ≤2.5 Gy/cell turnover of ^90^Sr (Figure 4A), ≤10 Gy/cell turnover of ^144^Ce (B), ≤11 Gy/cell turnover of ^91^Y (C) and ≤60 Gy/cell turnover of ^90^Y (D) also did not cause a significant increase in death when compared to controls (*p* < 0.05). Dogs exposed to ^90^Y received a much higher dose per cell turnover than those dogs exposed to the other radionuclides. This is due to the relatively short half-life of ^90^Y resulting in the total dose delivered in a single turnover.

## 3. Discussion

Carcinogenesis is a very complicated process and requires change in many key events in the critical pathways leading to cancer. These changes move normal cells towards a cancerous phenotype. It is also essential that the cancer cell progress and escape elimination by many other protective body processes such as the immune system [19] and selective radiation induced apoptosis [20]. Thus, it has been postulated that ‘it takes a tissue to make a cancer’ [12] and that changes in single cells such as mutations and chromosome aberrations in single cells are thought to be part of the process but may not be sufficient to induce cancer. Our data strongly support this observation. The mechanisms of cancer induction from a single or short term exposure seem to be different than those following protracted non-uniformly distributed radiation as seen from inhaled radioactive materials. For single whole body exposures, where all the protective mechanisms are exposed, mutations and chromosome aberrations seem to play an important role [15]. When the dose is protracted and non-uniformly distributed, many protective mechanisms are spared decreasing the risk per unit of dose. Dose protraction allows for more repair of molecular damage and the cancer response is very dependent on total tissue responses [12]. Cell killing and the resultant tissue proliferation, inflammatory responses, and tissue disorganization become the prime mechanisms involved in the production of cancer following protracted exposure from inhaled radionuclides. Radiation is a very efficient cell killer. Radiation induced transformed cells are killed via selective apoptosis [6]. Cell killing stimulates cell proliferation to replace the lost cells. It has been demonstrated in the liver that radiation induced damage from low LET radiation delivered at a low dose-rate followed by stimulation of cell proliferation increases the frequency of liver cancer [21], especially after large amounts of chromosome damage have been produced [10]. Recently, it has been suggested that each cell division increases the probability of a genetic alteration and that spontaneous cancer frequency may in part be explained by these ‘bad luck’ errors [14]. The stimulation of cell division through cell killing increases the number of cell divisions and could increase these errors and play a critical role in cancer induction. Radiation exposure also produces a dose related increase in chromosome aberrations [22,23] and mutations. The radiation induced mutations are primarily deletions and losses of genetic material [24]. Many of the radiation induced changes are lethal and do not lead to excess cancers. Thus, radiation is not an effective mutagen and is regulated accordingly [1,2].

The wide range of effective half-lives of the different radionuclides, from a few days for ^90^Y to almost two years for ^90^Sr, result in very different doses per cell turnover. The dose per cell turnover was calculated assuming that the average cell turnover time of the lung epithelial cells was 30 days. Combining the cell turnover time, the effective half-lives of each radionuclide, and the measured dose rates it was determined for ^90^Y all the dose was delivered in a single cell turnover and the dose was delivered over 7, 22, and 49 cell turnover for ^91^Y, ^144^Ce, and ^90^Sr, respectively. The dose per cell turnover had a wide range of values. The dose per cell turnover that resulted in increased cancer frequency from exposure to ^90^Y was 65–90 Gy/cell turnover and also a total dose of 65–90 Gy. At doses higher than this, acute lung disease resulted in marked life shortening with no increase in lung cancer. When the dose per cell turnover was further decreased to less than 65 Gy, no increase in lung cancers was observed in this group. This dose is very high when compared to the dose per cell turnover of other radionuclides. This is due to all of the dose of ^90^Y being delivered in a single cell turnover, thus cell death or cell cycle alterations due to cell division did not occur. The same evaluation for each of the radionuclides found that dose below 30 Gy/cell turnover of ^91^Y resulted in an increased frequency of lung cancer and doses below 15 Gy/cell turnover did not result in measured lung cancer. For ^144^Ce doses of greater than 10 Gy/cell turnover, the result was elevated lung cancer. For this radionuclide, there were several lung cancers produced following low total doses per cell turnover. These cancers may have been spontaneous and not related to dose or may indeed have been produced by the low doses per cell turnover. Because of the small numbers of animals in the study, the difference cannot be determined. With the longest lived radionuclide ^90^Sr, a dose of greater than 3 Gy/cell turnover resulted in increased lung cancer frequency. Again, at dose per cell turnover of less than 3 Gy/cell turnover no lung cancers were observed. These results support the argument that when delivered at a low dose rate radiation exposure may not cause a significant increase in cancer risk. The damage per cell turnover has to be high enough to result in significant cell killing, tissue disorganization, breakdown of cell/cell and cell/tissue communication, and the induction of a chronic inflammatory lung disease to result in a significant increase in lung cancer. When these conditions are present, the frequency of lung cancer is very high.

After exposure to high dose rates it has been established that radiation produces excess cancers. Using a LNT model the increase is described as resulting in an increase cancer frequency of 5%/Sv [1,2]. Since low doses and dose rates produce less cancer per unit of total dose, there is a need for a DDREF. The value to use for the DDREF has had extensive review and discussion and values for different biological endpoints which range from as low as 1.0 [25] to as high as 30 [6] exist. Major scientific organizations still have major discussions on both the risk estimates and the corrections needed to correct these values for low dose rates and non-uniform dose distribution [1,2,3,26]. The current manuscript demonstrates that cell killing, stimulation of cell proliferation, tissue disorganization, and chronic inflammatory disease are perhaps the most important factors for radiation induced lung cancer following low dose rate exposures to low LET radiation.

## 4. Materials and Methods

### 4.1. Animals

All the dogs used in these studies were produced and raised at the Inhalation Toxicology Research Institute (ITRI) using a randomized breeding program that helped to minimize genetic drift so that the dogs put into the experiments early in the studies were of a similar genetic background as those exposed later. This helped to minimize genetic variability in the experiment. For this manuscript, all data from these experiments was obtained from the Lovelace Institute Archive online database.

The methods for exposure, distribution, and dose and those used to determine cause of death in the animals have been carefully outlined and reviewed [4,27]. Briefly, the dogs were exposed by inhalation to different beta gamma emitting radionuclides (^90^Y, ^91^Y, ^144^Ce, and ^90^Sr) in fused aluminosilicate particles (FAP) which resulted in deposition and retention of the radioactive material in the lung and lung associated lymph nodes. Details of the exposure equipment and personnel protection procedures are carefully outlined in the ITRI annual report [27]. Since all the radionuclides were either pure beta emitters or beta gamma emitters, it was very easy to detect any radioactivity not properly contained. Briefly the animals were exposed in sealed chambers to the radioactive aerosol through a single nose only inhalation. There was a positive flow of air from the room into the chambers to prevent radioactive aerosol from getting into the exposure room. The radioactivity in the room was carefully monitored. All workers in the room wore full protective clothing with contained breathing air packs to protect against inhalation of radioactive material. After exposure, the dogs were placed in individual metabolism cages during clearance of radioactive material from the upper respiratory and GI tracts. When the secreted activity reached a low level dogs were moved into runs with two to three animals per run. Because of the short range of the beta particles there was little radiation dose delivered from one dog to his roommate or from the dogs to the animal handlers. With careful monitoring the potential for radiation exposure of the personnel was minimized.

Each dog was counted at regular intervals after exposure and the clearance of the radioactive material determined. These clearance curves were combined with the level of activity at sacrifice and the total dose and the changing dose rate to the respiratory tract derived. These radionuclides have very different physical half-lives and very similar biological half-lives. The dose and dose-rate varied over a wide range from days to years. For the radionuclides with a short physical half-life (^90^Y and ^91^Y) the effective half-life and dose rate primarily decreased as a function of the physical half-life. For the longer-lived radionuclides (^144^Ce and ^90^Sr) clearance and the biological half-life were important in the decrease of the effective half-life and dose rate.

Each dog was given routine scheduled physical exams and monitored for radiation induced changes over their life time. Changes in respiratory function were follow up with X-rays to determine the status of the lung morphology and function. Combining these techniques, it was possible to determine the time of onset of cancer and the degree of pulmonary injury. This information was recorded for each animal. This experimental design made it possible to treat each dog as a clinical subject and to construct dose-rate–response, time–response, and activity–response relationships for the cause of death for each animal. At death or sacrifice complete histopathological evaluation was done on each dog. The changes observed were classified using SNODOG, a modified version of Systematized Nomenclature of Medicine (SNOMED), the standardized database for all histopathology [27] and verified by two or more pathologists. Pulmonary function [28], changes in connective tissue types, and amount of collagen was measured [29] and the morphology and histopathological changes in the lung were used to determine the degree of pulmonary injury, fibrosis, chronic inflammatory disease, and radiation pneumonitis in each dog. The tumor types, time of onset, and numbers were documented. The cancer types were classified using SNODOG. All the cancers used in all this evaluation were malignant with the majority of them being lung carcinomas.

Since dose rate has been postulated to be the important variable in the production of cancer by internally deposited radioactive materials [9,10], it was important to carefully define this variable for the rapidly changing dose-rates produced by these internally deposited radioactive materials. It is important to apply the correct metric to relate dose-rate to biological effects. Raabe [9,10] calculated the dose rate by using the total dose to the animal divided by the time of death and used this to estimate the ‘dose rate’. This simple definition was useful in modeling but was not an accurate representation of the exposure patterns, damage per cell turnover, or potential for different number of cell populations to be exposed by these internally deposited radioactive materials. Our approach was to determine the dose rate at the time when half of the total dose was delivered and from this to estimate the dose delivered per cell turnover at this time. Thus, half the dose per cell turnover would be delivered at a higher dose-rate and half at a lower dose rate.

### 4.2. Dose Rate Calculations

#### Dose per Cell Turnover

The FAP resulted in the radioactivity being retained in the lung and the lung associated lymph nodes so that the majority of the dose was delivered to the lung epithelial cells. It has been well established that the induction and loss of damage in tissues can be dependent on cell turnover time and cell division. The accumulation of chromosome damage has been shown to be dependent on cell turnover. In the liver [8] and blood lymphocytes [22] where cell turnover times are long, the accumulation of chromosome damage is mostly dependent on total dose or dose to the cells being scored for damage. In rapidly dividing cells like bone marrow there is an equilibrium set up between induction of the damage as a function of dose rate and the loss of the damage through repair and cell division [7]. Therefore, the dose rate per cell turnover (DR_CT_) was calculated and used in this manuscript as the most useful metric of damage based on the average doubling time of a lung epithelial cells. This is very difficult since the lung has more than 30 different cell types and each of these have different cell turnover times [30]. The type II alveolar cells are thought to be the stem cells in the adult lung that maintain the integrity of the lung [31]. Early data on the cell kinetics of lung cells suggested that type II epithelial cells have a turnover time of about 30 days [32]. This still seems to be a good estimate of lung cell turnover without stress. However, it has been demonstrated that when there is lung injury that cell turnover times for type II cells become more rapid and they divide, differentiate, and replace damaged type I cells [30]. Other data also suggested that secretory cells can also be simulated to divide when the lung is injured [33] and may play a role in replacement of damaged cells. For this manuscript, it is assumed that at the dose rates where cell killing and lung injury were minimal the lung epithelial cell turnover time is 30 days and that the radiation delivered at a low dose rate from these internally deposited radioactive materials accumulates the damage in the same cell over this period. As the dose rate increases and the damage becomes more severe the cell turnover time will be shorter and the dose to the cells between cell divisions will be reduced. Since it has been demonstrated that chromosome damage and repair through cell division and other mechanisms sets up an equilibrium the cell turnover time is critical in understanding the influence of cell turnover time on the induction of tissue damage and cancer.

For ^91^Y, ^90^Sr, and ^144^Ce, the time to deliver 95% of the dose (*t*_95%_) was calculated (equal to 4.32 times the effective half-life). The total number of cell turnovers occurring during this dose deposition was calculated by dividing *t*_95%_ by 30 days. The dose rate was then calculated by dividing the cumulative dose at 95% deposition (*D*_95%_) by the number of cell turnovers, where

(1) $$DR_{CT}=\frac{D_{95\%}}{\frac{t_{95\%}}{30}}$$

In many of the animals inhaling high activity particles, the total lifespan was less than the time to deliver 95% of the dose. In these animals, the dose rate per cell turnover was calculated using the cumulative dose at death and the lifespan post exposure, where

(2) $$DR_{CT}=\frac{D_{d}}{\frac{t_{L}}{30}}$$

For ^90^Y, where *t*_95%_ is much less than 30 days (10.8 days), all of the dose was delivered within the first cell turnover. Therefore, the dose rate per cell turnover is the same as the cumulative dose at death, where

(3) $$DR_{CT}=D_{d}$$

### 4.3. Statistical Analysis

Dose rates were graphed and transformed to a log–log scale. Linear regression analysis was performed and slopes were considered significantly different from each other when *p* < 0.05. Kaplan–Meier curves were used to determine survival at different cell turnover doses. Significant differences were determined using Mantel–Cox test and Bonferroni corrections where *p* < 0.05. For cancer frequency analysis, χ^2^ tests were used to determine if cancer frequency of exposed groups (observed) were significantly different (*p* < 0.05) than control (expected). All statistical analyses were performed using GraphPad Prism software (San Diego, CA, USA).

## 5. Conclusions

Mutations, chromosome aberrations, and genomic instability may play a role in radiation induced cancer but at doses and dose rates where large numbers of aberrations and mutations would be produced there is no life shortening or increase in cancer frequency. This suggests that these events are of secondary importance and that tissue responses and damage produced by higher doses per cell turnover and total doses are major factors in producing lung cancer from internally deposited radioactive materials. When the radiation insult is at levels that do not induce these critical tissue changes, cancer frequency is not significantly increased. Cell killing, tissue disorganization, and chronic inflammatory disease are products of high dose per cell turnover, total dose, and dose rates leading to cancer. At lower levels of radiation insult these are not produced and as a result there is no development of cancer. The well-defined breaks in the survival curves, cancer incidence, and life span demonstrate that there is a very non-linear dose response relationship with real cancer and life span thresholds following inhalation of radioactive materials and do not support the LNT hypothesis. These observations require a paradigm shift away from the hit theory, single mutations, and cancer to a cell killing based paradigm which requires total tissue involvement during the induction of cancer.

## Figures and Tables

**Table 1:** The half-lives, time to deposit 95% of total dose of radionuclides, and average dose/cell turnover.

Radionuclide Infused Aluminosilicate Particles	Physical Half-Life	Effective Half-Life in Lung (days)	Average Time to Deliver 95% of Total Dose (days)	Average Number of Cell Turnovers *	Average Dose/Cell Turnover (Gy) *
^90^Sr	29 year	600	1473	49	26.65
^144^Ce	285 days	175	670	22	21.53
^91^Y	59 days	50	201	7	31.70
^90^Y	2.6 days	2.5	11	1	116.18

* Assuming a cell turnover time of 30 days.
